# Evolutionary and phylogenetic aspects of the chloroplast genome of *Chaenomeles* species

**DOI:** 10.1038/s41598-020-67943-1

**Published:** 2020-07-10

**Authors:** Jiahui Sun, Yiheng Wang, Yanlei Liu, Chao Xu, Qingjun Yuan, Lanping Guo, Luqi Huang

**Affiliations:** 1 National Resource Center for Chinese Materia Medica, China Academy of Chinese Medical Sciences, Beijing, 100700 China; 2 State Key Laboratory of Systematic and Evolutionary Botany, Institute of Botany, Chinese Academy of Sciences, Beijing, 100093 China; 3 University of Chinese Academy of Sciences, Beijing, 100049 China

**Keywords:** Phylogenetics, Plant evolution

## Abstract

*Chaenomeles* (family Rosaceae) is a genus of five diploid species of deciduous spiny shrubs that are native to Central Asia and Japan. It is an important horticultural crop (commonly known as flowering quinces) in Europe and Asia for its high yield in fruits that are rich in juice, aroma, and dietary fiber. Therefore, the development of effective genetic markers of *Chaenomeles* species is advantageous for crop improvement through breeding and selection. In this study, we successfully assembled and analyzed the chloroplast genome of five *Chaenomeles* species. The chloroplast genomes of the five *Chaenomeles* species were very similar with no structural or content rearrangements among them. The chloroplast genomes ranged from 159,436 to 160,040 bp in length and contained a total of 112 unique genes, including 78 protein-coding genes, 30 tRNAs, and 4 rRNAs. Three highly variable regions, including *trnR-atpA*, *trnL-F*, and *rpl32-ccsA*, were identified. Phylogenetic analysis based on the complete chloroplast genome showed that *Chaenomeles* forms a monophyletic clade and had a close relationship with the genera *Docynia* and *Malus.* Analyses for phylogenetic relationships and the development of available genetic markers in future could provide valuable information regarding genetics and breeding mechanisms of the *Chaenomeles* species.

## Introduction

The genus *Chaenomeles* Lindley belongs to the tribe Maleae and is an ecologically and economically important part of the Rosaceae family^1^. *Chaenomeles* is closely related to the well-known fruit crop genera *Cydonia* (quince), *Malus* (apple), and *Pyrus* (pear). It comprises of five diploid (2n = 34) species: one species is endemic to Japan, and four originate from central Asia. Cultivation *Chaenomeles* plants as horticultural crops has been initiated in Europe and in Asia^2,3^.

*Chaenomeles japonica* (Thunb.) Lindl. ex Spach (Japanese quince) is a dwarf shrub that grows in central and south Japan, and is strongly self-incompatible that encourages outcrossing^4^. *C. speciosa* (Sweet) Nakai (flowering quince) is a large shrub (2–5 m) that grows at an altitude of 200–1,700 m in central and southern China, Tibet and Burma, and is traditionally used in medicines^5^. *C. cathayensis* (Hemsl.) Schneider (Chinese quince) is a large shrub or small tree (up to 6 m) that grows at an altitude of 900–2,500 m in southern China, Bhutan and Burma. *C. speciosa* and *C. cathayensis* are sympatric in the province of Yunnan, China^6^. *C. thibetica* Yü (Tibetan quince), is a large shrub that grows in Tibet and western Sichuan^6^. *C. sinensis* (Dum.Cours.) Koehne, also referred as *Pseudocydonia sinensis* (Chinese quince), is a shrub or small tree (5–10 m) that grows in central and southern China. Three of these species (*C. cathayensis*, *C. japonica*, and *C. speciosa*) have been used to create several interspecific hybrids for approximately 400 years, resulting in more than 500 cultivars^3^, with the aim of developing new ornamental cultivars.

There were less genetic information of *Chaenomeles* in public database. Understanding the genetic diversity among and within wild populations of *Chaenomeles* was effective for plant breeding and the development of ex situ conservation strategies for plant genetic resources. Isozymes, RAPDs, and several chloroplast genome markers have been used in population genetics studies^2,7–9^. However, these markers have low variation and reproducibility. Therefore, there is need to develop effective genetic markers to facilitate the identification, conservation, utilization and breeding of *Chaenomeles* species.

The chloroplast genome has a stable structure that conserves the size and gene content^10^. The chloroplast genome of most angiosperm plants is composed of two inverted repeats (IR), which separate the large (LSC) and the small (SSC) single copy regions. Furthermore, the size of a typical angiosperm chloroplast genome ranges from 115 to 165 kb and contain 110–130 genes, with about eighty protein-coding genes, four rRNA genes and thirty tRNA genes^11,12^. Complete chloroplast genome sequences have been widely used as a source of valuable data for understanding evolutionary biology^13–16^. For example, chloroplast genome data have been used extensively for plant phylogenetic analyses at family/genus/species levels and DNA chloroplast barcoding for accurate identification of plant species^17–19^. The development of DNA sequencing technology has resulted in the extensive use of chloroplast genomes for species identification and molecular phylogenetic studies.

In this study, we sequenced the chloroplast genome for the five species of *Chaenomeles* and a closely related species, *Docynia delavayi*. The specific aims of this study were to (1) understand the conservation and diversity of *Chaenomeles* chloroplast genome through comparative genomic approaches; (2) identify the most variable regions of these chloroplast genomes as DNA barcodes for future species identification and phylogeny studies for the species and genera of Rosaceae; and (3) determine their phylogenetic relationships using the chloroplast genome sequence data.

## Materials and methods

### Plant materials and DNA extraction

Fresh young leaves of *C. cathayensis*, *C. japonica*, and *C. sinensis* from Beijing Botanical Garden, Beijing (China), *C. thibetica* from Bomê County, Tibet (China), and *D. delavayi* from Kunming Institute of Botany, Yunnan (China) were obtained and subsequently dried with silica gel. Voucher specimens were deposited at the PE herbarium of the Institute of Botany, Chinese Academy of Sciences. The species’ DNA was extracted with a DNeasy Plant Mini Kit (Qiagen Co., Germany). The quality and quantity of the genomic DNA were measured on 1% agarose gel and by using a Thermo Scientific NanoDrop.

### Illumina sequencing, assembly, and annotation

Purified DNA was used to generate short-insert (350 bp) paired-end sequencing libraries according to the Illumina standard protocol. The entire genome sequencing was carried out using a HiSeq X Ten system (Novogene, Beijing). Approximately 5 GB of raw data were generated from each genome with 150 bp paired-end read lengths.

Low-quality reads and adapters were filtered from the raw data by using Trimmomatic^20^. The clean paired-end reads were qualitatively assessed and assembled with SPAdes 3.6.1^21^. The contigs were then checked using BLAST searches against the available complete chloroplast sequence of *C. speciosa* (KT932965). The relative position and direction of each contig were manually adjusted with Sequencher 5.4.5 according to the reference genome. Chloroplast genome annotation was performed with Plann^22^ using the *C. speciosa* reference sequence from Genbank. The annotated chloroplast genome sequences were submitted to GenBank under accession numbers MN506259–MN506262, and MN506264. A gene map of the annotated *Chaenomeles* chloroplast genome was drawn online using OGdraw^23^.

### Genome comparison

To investigate the divergence in the chloroplast genome, the identity across the whole complete chloroplast (cp) genome was visualized using the mVISTA program for the five species, with the *C. speciosa* genome from GenBank as the reference. Default parameters were utilized to align the chloroplast genomes in Shuffle-LAGAN mode, and a sequence conservation profile was visualized using a mVISTA plot^24^. Any large structural events, such as gene order rearrangements and IR expansions/contractions, were recorded.

All five plant species’ chloroplast genomes were aligned using MAFFT v7^25^, followed by an adjustment with Se-Al 2.0^26^. To elucidate the level of sequence variation, SNP variation and k2p-distance among *Chaenomeles* chloroplast genomes were calculated using MEGA 6.0 software^27^.

To explore the diverging hotspot regions in *Chaenomeles* species and facilitate their utilization in identification, sliding window analysis was conducted to generate the nucleotide diversity of the chloroplast genome using the DnaSP v5.10 software^28^. The step size was set to 200 bp, with an 800 bp window length.

### Analysis of tandem repeats and single sequence repeats

The REPuter program^29^ was used to identify repeats: forward, reverse, palindrome, and complement sequences. The following settings for repeat identification were used: (1) hamming distance equal to 3; (2) minimal repeat size set to 30 bp; and (3) maximum computed repeats set to 90 bp. Tandem repeats were identified using the web-based Tandem Repeats Finder (https://tandem.bu.edu/trf/trf.html), with 2, 7, and 7 set for the alignment parameters match, mismatch, and indel, respectively. Simple sequence repeats (SSRs) were detected using GMAT^30^ with thresholds of ten repeat units for mononucleotide SSRs, five repeat units for dinucleotide SSRs, four repeat units for trinucleotide SSRs, and three repeat units for tetra-, penta-, and hexa-nucleotide SSRs.

### Phylogenetic reconstruction

We downloaded 28 published chloroplast genomes of Maleae from Genbank that were included in the analyses as the outgroup taxa to perform the phylogenetic reconstruction. A total of 34 chloroplast genomes were aligned using MAFFT v7^25^. The gaps in the alignment were stripped. Phylogenetic trees were constructed using maximum likelihood (ML) and Bayesian analysis (BI) methods. The phylogenetic analyses used the best-fitting models of nucleotide substitution selected in ModelFinder^31^ under the Bayesian information criterion. The maximum likelihood (ML) analyses were performed in RAxML v.8.1.24^32^. The support branches (BS) were assessed with 1,000 rapid bootstrapping replicates. Bayesian inference was performed using MrBayes v3.2.2^33^. The Markov chain Monte Carlo (MCMC) analysis was run for 2 × 5,000,000 generations. The fist 25% of the trees corresponding to the “burn-in” period were discarded, and the remaining tree parts were used to construct the majority-rule consensus tree. The stationarity series was considered to be reached when the average standard deviation of the split frequencies remained < 0.01.

## Results and discussion

### Chloroplast genomes features of *Chaenomeles* species

After Illumina paired-end sequencing, 25,235,314–28,277,676 reads were obtained for the five *Chaenomeles* species. Through de novo assembly, contig selection and second reference based assembly were then generated for the five complete chloroplast genomes. The assembled chloroplast genome of the five examined species had a high coverage depth of about 3,000×.

**Figure 1:**
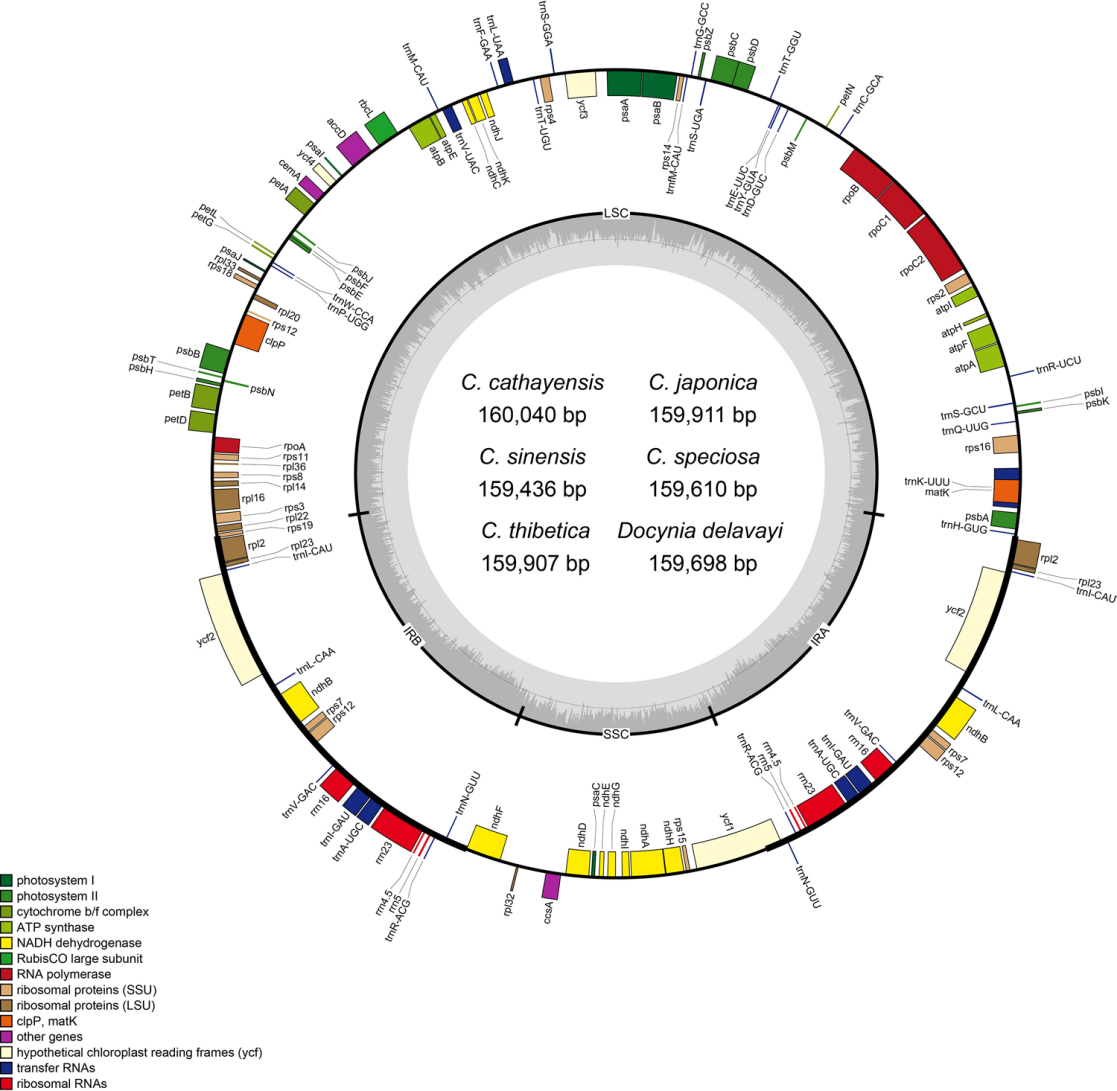
Chloroplast genomes of *Chaenomeles*. Genes on the inside are transcribed in a clockwise direction, while genes on the outside are transcribed in a counterclockwise direction.

The complete chloroplast genomes of the five *Chaenomeles* species ranged from 159,436 bp (*C. sinensis*) to 160,040 bp (*C. cathayensis*) in length. All of the *Chaenomeles* chloroplast genomes displayed the typical quadripartite structure of angiosperm cpDNA (Fig. 1, Table 1), which consists of a pair of IR regions (26,300–26,393 bp) separated by a LSC region (87,476–87,937 bp) and a SSC region (19,229–19,345 bp). The overall guanine-cytosine (GC) content was 36.5–36.7%, indicating nearly identical levels among the *Chaenomeles* chloroplast genomes. GC content in the LSC, SSC and IR regions were 34.3–34.4%, 30.2–30.5% and 42.6–42.7%, respectively. The high GC content in the IR regions is due to the reduced presence of AT nucleotides in the four duplicate rRNA genes (rrn16, rrn23, rrn4.5, and rrn5). The GC content of the *Chaenomeles* chloroplast genome is close to that reported for other Rosaceae chloroplast genomes^34,35^. The *Chaenomeles* chloroplast genomes were compared to previously published data and showed highly similarity in genome structure^34,36,37^. With regard to the genome size, the length of complete chloroplast genome varies from 147 to 163 kb across Rosaceae^34^. The main reason for variation in genome length was expansions and contractions in IR regions and intergenic regions. In *Chaenomeles*, the junctions of IR and LSC or SSC have less variations, and exhibit the typical Rosaceae genome structure^36^.

**Table 1:** Summary chloroplast genome features of five *Chaenomeles* species and *Docynia delavayi*.

Gene features	*C. cathayensis*	*C. japonica*	*C. sinensis*	*C. speciosa*	*C. thibetica*	*Docynia delavayi*
Accession number in Genbank	MN506260	MN506261	MN506262	KT932965	MN506264	MN506259
Total cpDNA size (bp)	160,040	159,911	159,436	159,610	159,907	159,698
LSC length (bp)	87,937	87,814	87,476	87,781	87,851	87,804
SSC length (bp)	19,345	19,311	19,246	19,229	19,298	19,156
IR length (bp)	26,379	26,393	26,357	26,300	26,379	26,369
Total GC content (%)	36.5	36.6	36.7	36.6	36.6	36.6
LSC GC content (%)	34.3	34.3	34.4	34.3	34.3	34.3
SSC GC content (%)	30.2	30.3	30.5	30.3	30.3	30.4
IR GC content (%)	42.6	42.6	42.7	42.6	42.6	42.7
Total number of genes	112	112	112	112	112	112
Protein-coding genes	78	78	78	78	78	78
rRNA genes	4	4	4	4	4	4
tRNA genes	30	30	30	30	30	30

The *Chaenomeles* chloroplast genome contained a total of 112 unique genes, including 78 protein-coding genes, 30 tRNAs, and four rRNAs. Nineteen genes were duplicated in the IR, including eight protein-coding genes, seven tRNA genes and four rRNA genes. Fifteen distinct genes had a single intron, and two genes (*ycf3* and *clpP*) had two introns. The *rps12* gene is trans-spliced with the 3′exon being duplicated in the IR, while the 5′ end is located at the LSC region. *TrnK-UUU* had the largest intron (2,561–2,570 bp) containing the *matK* gene. Compared with other Rosaceae chloroplast genomes, the number of unique genes were conserved^35,36,38,39^, and no significant changes of gene orders were found in the *Chaenomeles.*

### Comparative analysis of the *Chaenomeles* plastomes

**Figure 2:**
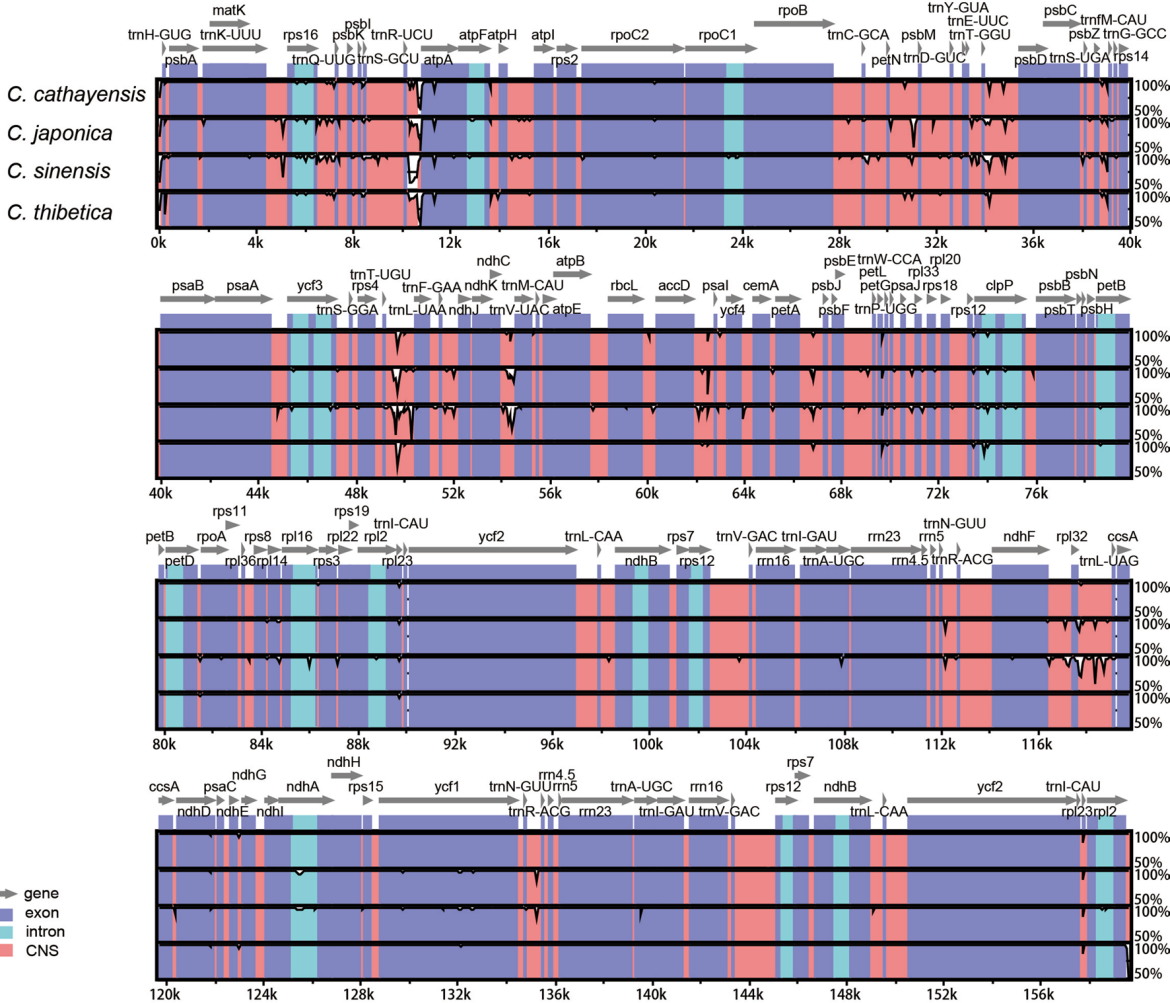
Identity plot comparing the chloroplast genomes of the five *Chaenomeles* species, using *C. speciosa* as a reference sequence. Genome regions are color coded as protein-coding, rRNA, tRNA, intron, and conserved non-coding sequences (CNS).

The mVISTA program was used to analyze the overall sequence identity of the chloroplast genome of the five *Chaenomeles* species, using the annotation for *C. speciosa* as a reference (Fig. 2). The *Chaenomeles* chloroplast genome displayed similar structure and gene order. The divergence level of the non-coding regions was higher than that of the coding regions. In addition, LSC and SSC regions had a larger divergence than the IR regions. A higher sequence divergence was found in the single copy regions than in the IRs and in the non-coding regions than in the coding regions, which is in accordance with the results found for other taxa^40–42^.

To further understand the chloroplast genome sequence divergence among *Chaenomeles* species, the number of nucleotide substitutions and sequence k2p-distances were designated to represent the level of divergence. The five *Chaenomeles* chloroplast genomes were fully aligned, giving an alignment matrix of 161,903 bp; 843 variable sites and 147 information sites were found. The number of nucleotide substitutions in pairwise comparisons between the five species ranged from 63 to 695, and the k2p-distances ranged from 0.0004 to 0.0041 (Table 2). The lowest sequence divergence was between *C. cathayensis* and *C. thibetica*, while the highest sequence divergence occurred between *C. sinensis* and *C. speciosa*.

**Table 2:** Numbers of nucleotide substitutions and sequence distances in five *Chaenomeles* complete chloroplast genomes.

	*C. cathayensis*	*C. japonica*	*C. sinensis*	*C. speciosa*	*C. thibetica*
*C. cathayensis*		282	633	695	63
*C. japonica*	0.0018		588	304	284
*C. sinensis*	0.0040	0.0037		651	631
*C. speciosa*	0.0006	0.0019	0.0041		103
*C. thibetica*	0.0004	0.0018	0.0040	0.0006	

The upper triangle shows the number of nucleotide substitutions. The lower triangle indicates the number of sequence distances in complete chloroplast genomes.

**Figure 3:**
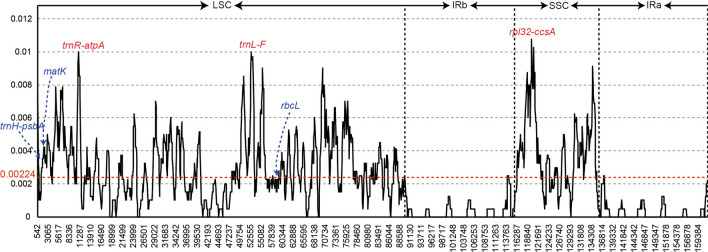
Nucleotide diversity of the *Chaenomeles* chloroplast genomes.

To identify the sequence divergence hotspots, the nucleotide diversity (pi) value within the slide window of 600 bp was calculated (Fig. 3). The pi value in the windows varied from 0 to 0.01075, with a mean of 0.00224. Three highly variable regions (pi > 0.01), including *trnR-atpA*, *trnL-F*, and *rpl32-ccsA*, were identified in the *Chaenomeles* chloroplast genomes. Among these regions, *trnR-atpA* and *trnL-F* were located in the LSC region, and *rpl32-ccsA* was in the SSC region. All nucleotide diversity values in the IR regions were less than 0.003 and no highly divergent sequences were found; therefore, these regions were considered to be conserved, whereas the universal DNA barcodes (*matK, rbcL* and *trnH-psbA*) had lower pi values. We compared these three highly variable markers in more detail (Table 3). The aligned length of the markers ranged from 816 bp for *trnL-F* to 1,603 bp for *rpl32-ccsA*. *rpl32-ccsA* showed the highest number of variable and informative sites. The average nucleotide diversity of the three rapidly evolving regions was 0.00986, which was 2.6 times higher than that of the universal DNA barcodes. The pi values of these regions showed 0.00373 (Table 3).

**Table 3:** Variability of nine variable markers and universal chloroplast DNA barcodes (*rbcL* and *matK*) in *Chaenomeles.*

Markers	Length	Variable sites		Information sites		Nucleotide diversity
		Numbers	%	Numbers	%	
*trnR-atpA*	1,142	26	2.28	3	0.26	0.01000
*trnL-F*	816	18	2.21	4	0.49	0.01000
*rpl32-ccsA*	1603	30	1.87	5	0.31	0.00969
*trnR-atpA*+*trnL-F*+*rpl32-ccsA*	3,561	74	2.08	12	0.34	0.00986
*rbcL*	1,427	9	0.63	0	0.00	0.00273
*matK*	1512	14	0.93	3	0.20	0.00410
*trnH-psbA*	354	4	1.13	2	0.56	0.00669
*rbcL*+*matK*+*trnH-psbA*	3,293	27	0.82	5	0.15	0.00373

Chloroplast genome markers are extensively used in plant phylogenetic studies to analyze relatedness and classify species. Some universal chloroplast regions, such as *rbcL*, *matK*, *ndhF*, *trnH-psbA*, *psbK-psbI,* and *atpB-rbcL*, have been used as markers in phylogenetic studies^13,43–45^. However, an increased number of studies have shown that it is inappropriate to use the universal markers to classify closely related species as they have lower variability. Comparative chloroplast genome analysis was a new strategy to identify the mutation hotspot markers^13,41^. The intergenic spacer *trnL-F* have a long time of use in plant phylogenetic and species identification studies^46,47^. In some groups this region often contains ploy A and T structures^43^ and affect sequence quality. The *rpl32-ccsA* marker includes two intergenic spacers (*rpl32-trnL* and *trnL-ccsA*) in the SSC region. More papers showed this region had higher variable sites^44^. *trnR-atpA* is less commonly used to reconstruct phylogenetic relationships or as DNA barcode. The highly variable makers discovered in this study could be regarded as potential molecular resources for species identification and applied in phylogenetic analyses of Rosaceae.

### Analysis of repeat elements

Repetitive sequences in the chloroplast genome play an important key role in the genome rearrangement and stabilization, and they provide important information for understanding the evolutionary history of plant species and sequence divergence^48–50^. SSRs or microsatellites, and dispersed long repeats were the two main motifs in the chloroplast genomes.

SSRs are important co-dominant molecular markers for evaluating germplasm, establishing phylogenetic and evolutionary relationships^51^, and they are widely present in the chloroplast genome^41^. Using GMAT analysis, mono-, di-, trin-, tetra-, penta-, and hexa-nucleotide SSRs were detected in every species, and each *Chaenomeles* chloroplast genome was found to contain 91 (*C. thibetica*) to 94 (*C. cathayensis*) SSRs. The number of SSRs are slightly lower than those reported in previous Rosaceae chloroplast genome studies with *Hagenia* (172)^36^ and *Rubus* (116)^37^.

**Figure 4:**
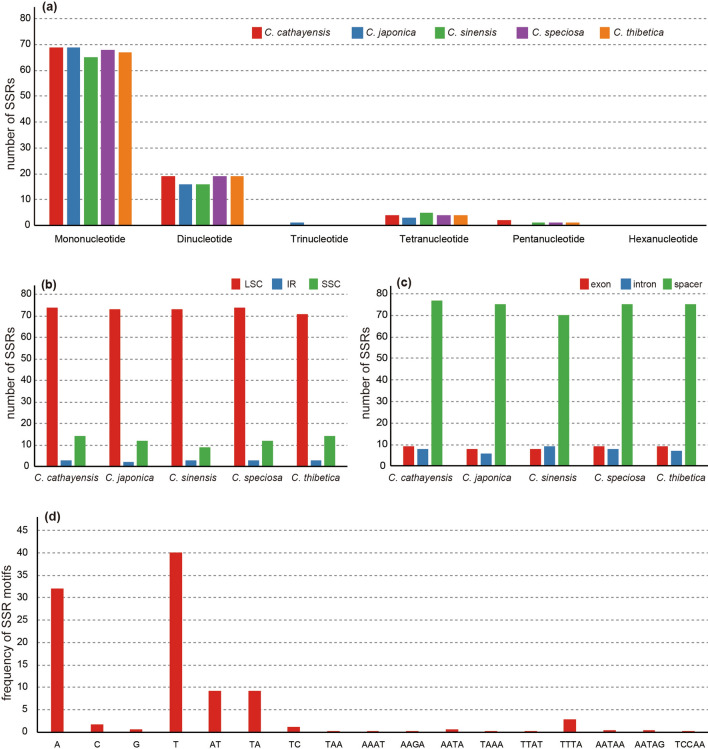
SSR loci analysis of five *Chaenomeles*. (**a**) Number of different SSRs types; (**b**) number of SSRs in spacer, exon, and intron; (**c**) number of SSRs in LSC, SSC, and IR regions; (**d**) frequency of identified SSR motifs in the different repeat classes.

All five *Chaenomeles* chloroplast genome had five types of SSRs, excluding the hexanucleotide SSR (Fig. 4a). In the five species examined, most of the SSRs were mononucleotide SSR (73.40%, 77.53%, 74.71%, 73.91%, and 73.63% in *C. cathayensis*, *C. japonica*, *C. sinensis*, *C. speciosa*, *C. thibetica*, respectively). SSRs were distributed more widely throughout the chloroplast genomes, and were usually located in the LSC regions (78.02–83.90%, Fig. 4b). Most of the SSRs were found in spacer regions (80.46–84.27%, Fig. 4c); only a few were located in the coding regions.

Almost all of the mononucleotide repeat sequences were comprised of A/T repeats (72.19%). Meanwhile, AT/TA repeats were the most common among dinucleotide SSRs (94.38%). In addition, two pentanucleotide repeats (AATAG and AATAG) were found in *C. cathayensis* and one in *C. speciosa* (AATAA), *C. sinensis* (TCCAA), and *C. thibetica* (AATAG) using our search criterion (Fig. 4d). In general, chloroplast genome sequences are highly conserved at the genus level, and in silico development of SSRs in chloroplast genomes has supported them as potentially transferable markers among species^52,53^. In addition SSRs are highly polymorphic and have been potential markers for establishing molecular evolutionary histories and demographic diversity^54,55^.

**Figure 5:**
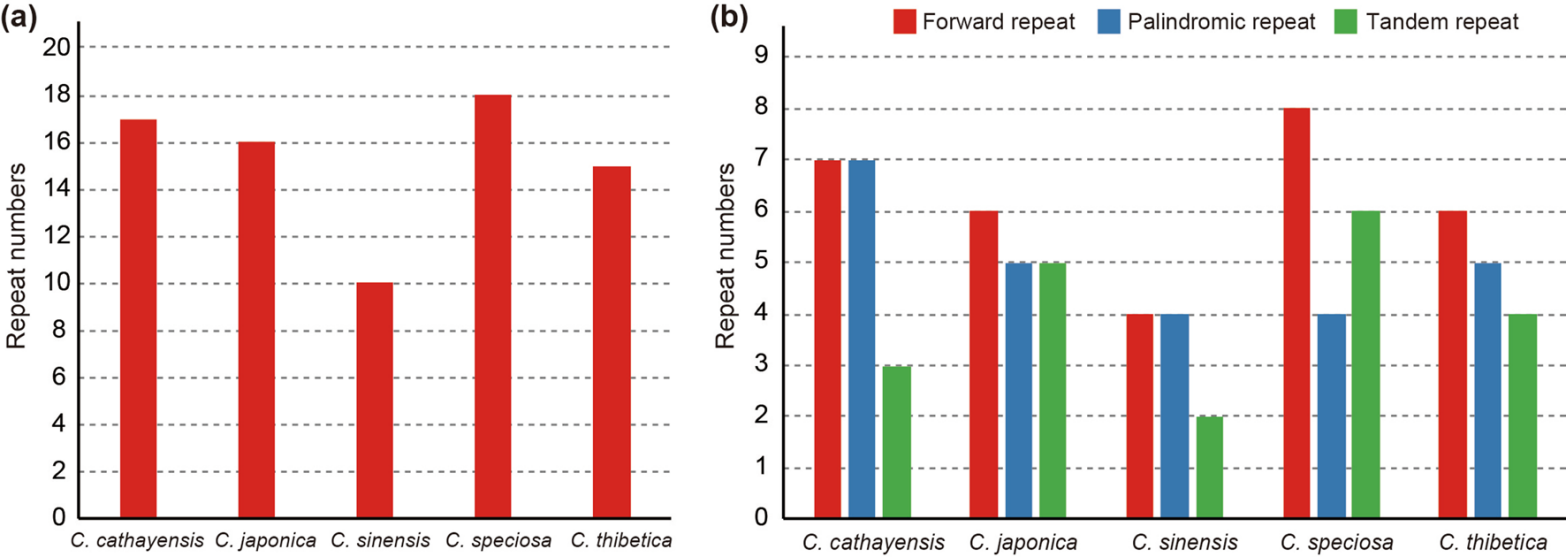
Long repeat sequences in the chloroplast genomes of five *Chaenomeles*. (**a**) Number of repeats; (**b**) Number of different repeats types.

We classified sequence dispersed repeat motifs into five categories: forward, reverse, palindrome, complement and tandem repeats. In the *Chaenomeles* chloroplast genome, we identified three repeat motifs (Fig. 5). In general, the forward repeats were the most common, except for *C. cathayensis* and *C. sinensis* that had as many forward repeats as palindromic repeats. In total, 76 repeats with more than 30 bp were detected in the five *Chaenomeles* chloroplast genome. *C. speciosa* contained the most repeats (18) compared to the other four species (10, 15, 16, and 17 repeats, respectively, Fig. 5). The majority of repeats (63.16%) ranged in size from 31 to 35 bp. The longest repeat was a forward repeat of 82 bp in *C. japonica*. Repeat sequences are considered to play an important role in genome recombination and rearrangement and also have phylogenetic information in some groups^50,56^.

### Phylogenomic analysis

Chloroplast genomes contain an abundance of phylogenetic information, which has been widely used for phylogeny reconstruction at different taxonomic levels, such as order, family, genus, and species in plants. Using chloroplast genome data, long-standing controversies related to various phylogenetically difficult groups have been resolved, supporting its importance in systematic studies.

**Figure 6:**
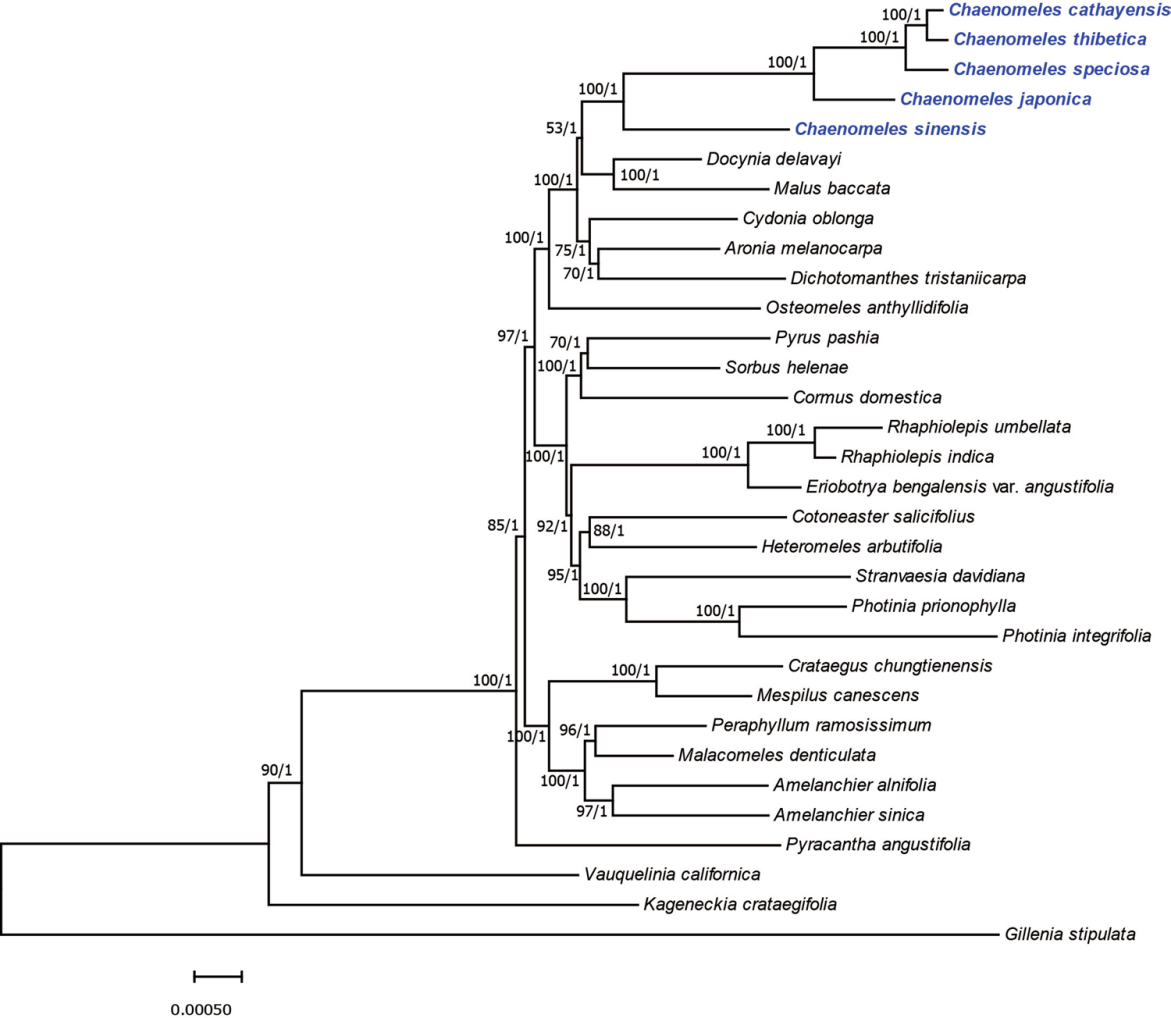
Phylogenetic tree reconstruction of 32 taxa using maximum likelihood and Bayesian inference methods based on the complete chloroplast genome sequences. Number of the branches indicate ML bootstrap support value/Bayesian posterior probability.

To better determine the phylogenetic position of *Chaenomeles* and further clarify the evolutionary relationships within the tribe Maleae, phylogenetic analyses was constructed based on the 32 Maleae complete chloroplast genomes, using *Gillenia stipulata* as an outgroup. The phylogenetic topologies of the ML and BI method were similar (Fig. 6), and most nodes were supported by high values (> 95%). However, some internal nodes tended to have poorer bootstrap support, indicating rapid radiation and/or incomplete lineage sorting. *Chaenomeles* was observed to be a sister lineage of *Docynia* and *Malus* based on low bootstrap support and posterior probability values (ML bootstrap support, BS = 53; posterior probability, PP = 1). The monophyly of *Chaenomeles* was strongly supported (BS = 100%, PP = 1). *C. sinensis* was the basal species in *Chaenomeles. C. sinensis* was once treated as a monotypic genera *Pseudocydonia*^57^. However, the morphological data and several chloroplast markers and ITS data^57^^,^^58^ did not support *Pseudocydonia* separated out of *Chaenomeles.* The result indicates the necessity of revising taxonomic boundaries of *Chaenomeles* and redefining taxonomic status of *C. sinensis*. The chloroplast genome phylogeny showed *C. cathayensis* was a sister species of *C. thibetica*. This results was congruent with Bartish et al.’s results, which recognized that *C. thibetica* appeared to be rather closely related to *C. cathayensis* using RAPDs and isozymes methods.

## Conclusions

In this study, we sequenced the total chloroplast genome of five *Chaenomeles* species by de novo sequencing, and showed that the chloroplast genome structure is well conserved throughout the genus. The comparative analyses revealed extremely low levels of sequence variability. However, repeat sequences, SSRs, and highly polymorphic regions were identified to be suitable for possible genetic markers. These markers could be considered for phylogenetic analysis and to resolve taxonomical discrepancies in *Chaenomeles* and potentially in other Rosaceae. Phylogenetic reconstruction based on the complete chloroplast genomes revealed the relationships among the five species of *Chaenomeles*. In summary, this study will be helpful for further research on the molecular evolution and speciation of this genus.

## Supplementary information

Supplementary file1 (DOCX 28 kb)

## Data Availability

The complete chloroplast sequence generated and analyzed during the current study are available in GenBank (MN506259–MN506262, and MN506264).
