# Supplementary material for: Evolutionary and phylogenetic aspects of the chloroplast genome of Chaenomeles species
Source: Sci Rep. 2020 Jul 10;10:11466. doi: 10.1038/s41598-020-67943-1 (PMC7351712; doi:10.1038/s41598-020-67943-1)
Supplement: Supplementary file 1 — Supplementary file1 (DOCX 28 kb) [file 41598_2020_67943_MOESM1_ESM.docx]

**Evolutionary and phylogenetic aspects of the chloroplast genome of *Chaenomeles* species**

Jiahui Sun^1,†^, Yiheng Wang^1,†^, Yanlei Liu^2,3^, Chao Xu^2^, Qingjun Yuan^1, *^, Lanping Guo^1, *^, Luqi Huang^1,*^

^1^ National Resource Center for Chinese Materia Medica, China Academy of Chinese Medical Sciences, Beijing 100700, China

^2^ State Key Laboratory of Systematic and Evolutionary Botany, Institute of Botany, Chinese Academy of Sciences, Beijing 100093, China

^3^ University of Chinese Academy of Sciences, Beijing 100049, China

†These authors contributed equally to this work.

*****Correspondence: yuanqingjun@icmm.ac.cn (Q.Y.); glp01@126.com (L.G.); huangluqi01@126.com (L.H.)

Table S1. List of genes found in the *Chaenomeles* chloroplast genome.

Table S2. List of chloroplast genome sequences used for phylogenetic analysis.

Table S1. List of genes found in the *Chaenomeles* chloroplast genome.

| **Category for genes** | **Group of gene** | **Name of gene** |
| --- | --- | --- |
| Photosynthesis related genes | Rubisco | *rbcL* |
|  | Photosystem Ⅰ | *psaA,psaB,psaC,psaI,psaJ* |
|  | Assembly/stability of photosystem Ⅰ | **ycf3,ycf4* |
|  | Photosystem Ⅱ | *psbA,psbB,psbC,psbD,psbE,psbF,psbH,psbI,psbJ,psbK,psbL,psbM,psbN,psbT,psbZ* |
|  | ATP synthase | *atpA, atpB, atpE, *atpF, atpH, atpI* |
|  | cytochrome b/f compelx | *petA, *petB, *petD, petG, petL, petN* |
|  | cytochrome c synthesis | *ccsA* |
|  | NADPH dehydrogenase | **ndhA, *ndhB, ndhC, ndhD, ndhE, ndhF ,ndhG, ndhH, ndhI, ndhJ, ndhK* |
| Transcription and translation related genes | transcription | *rpoA, rpoB, *rpoC1, rpoC2* |
|  | ribosomal proteins | *rps2, rps3, rps4, rps7, rps8, rps11, *rps12, rps14,rps15, *rps16, rps18, rps19,*rpl2, rpl14, *rpl16, rpl20, rpl22, rpl23, rpl32, rpl33,rpl36* |
|  | translation initiation factor | *infA* |
| RNA genes | ribosomal RNA | *rrn5, rrn4.5, rrn16, rrn23* |
|  | transfer RNA | **trnA-*UGC*, trnC-GCA, trnD-GUC, trnE-UUC, trnF-GAA,trnG-GCC, *trnG-UCC, trnH-GUG, trnI-CAU, *trnI-GAU,*trnK-UUU, trnL-CAA, *trnL-UAA, trnL-UAG, trnfM-CAUI,trnM-CAU, trnN-GUU, trnP-UGG, trnQ-UUG,trnR-ACG, trnR-UCU, trnS-GCU, trnS-GGA, trnS-UGA, trnT-GGU,trnT-UGU, trnV-GAC, *trnV-UAC, trnW-CCA, trnY-GUA* |
| Other genes | RNA processing | *matK* |
|  | carbon metabolism | *cemA* |
|  | fatty acid synthesis | *accD* |
|  | proteolysis | **clpP* |
| Genes of unknown function | conserved reading frames | *ycf1, ycf2,* |

Table S2. List of chloroplast genome sequences used for phylogenetic analysis.

| Organism | Accession |
| --- | --- |
| Amelanchier alnifolia | KY419978 |
| Amelanchier sinica | KY419998 |
| Aronia melanocarpa | KY420007 |
| Cormus domestica | KY419956 |
| Cotoneaster salicifolius | KY419943 |
| Crataegus chungtienensis | KY419947 |
| Cydonia oblonga | KX499857 |
| Dichotomanthes tristaniicarpa | KY420031 |
| Eriobotrya bengalensis var. angustifolia | KY419922 |
| Gillenia stipulata | KY419996 |
| Heteromeles arbutifolia | KY419965 |
| Kageneckia crataegifolia | KY420027 |
| Malacomeles denticulata | KY419982 |
| Malus baccata | KX499859 |
| Mespilus canescens | KY420022 |
| Osteomeles anthyllidifolia | KY419940 |
| Peraphyllum ramosissimum | KY420011 |
| Photinia integrifolia | KY419933 |
| Photinia prionophylla | KY419946 |
| Pyracantha angustifolia | KY419957 |
| Pyrus pashia | KY419974 |
| Rhaphiolepis indica | KY419927 |
| Rhaphiolepis umbellata | KY419931 |
| Sorbus helenae | KY419924 |
| Vauquelinia californica | KY419925 |
